# Explainable AI for public health surveillance: investigating the persistent crisis of intentional injury mortality (suicide and homicide) in the Americas

**DOI:** 10.1038/s41598-026-51327-y

**Published:** 2026-05-24

**Authors:** Sherin Kularathne, Namal Rathnayake, Ruwan Jayathilaka, Iori Nakaoka, Yukinobu Hoshino

**Affiliations:** 1 School of Data and Innovation, Kochi University of Technology, 2-22 Eikokuji-cho, Kochi, 780-8515 Kochi Japan; 2 Advanced Institute for Marine Ecosystem Change (WPI-AIMEC), Yokohama, 236-0001 Japan; 3 SLIIT Business School, Sri Lanka Institute of Information Technology, New Kandy Road, Malabe, Sri Lanka; 4 Faculty of Data Science, Shimonoseki City University, 2 Chome-1-1 Daigakucho, Shimonoseki, 751-8510 Japan

**Keywords:** Intentional injury mortality, Suicide, Homicide, Inflation, Economic growth, Corruption, Explainable artificial intelligence, Complex networks, Complex networks, Geography, Geography

## Abstract

Intentional injury mortality (IIM), comprising homicide and suicide, remains a critical public health crisis in the Americas, which not only has the highest regional homicide rates globally but is also the only region where suicide rates continue to rise. This study employs explainable artificial intelligence (XAI) to examine the structural and temporal drivers of IIM across 25 countries, based on data from the previous two decades. Two complementary models were developed: a snapshot model based on contemporaneous socioeconomic indicators and a persistence-aware model incorporating lagged effects of predictors. Analyses were conducted across both income-level categories and geographic sub-regions to uncover context-specific patterns. While both models performed at acceptable levels in distinguishing immediate and enduring effects, persistence-aware models consistently outperformed snapshot models, thereby reframing IIM as a temporally sustained phenomenon. Feature importance, interpreted through SHapley Additive exPlanations (SHAP), highlighted the varying impacts of unemployment, inflation, corruption, and economic growth across income tiers and sub-regions. The results demonstrate that a combination of short-term shocks and the long-standing effects of governance and social factors drives IIM in the Americas. These findings underscore the need for dual-horizon policy approaches that address both immediate crises and structural root causes.

## Introduction

**Fig. 1:**
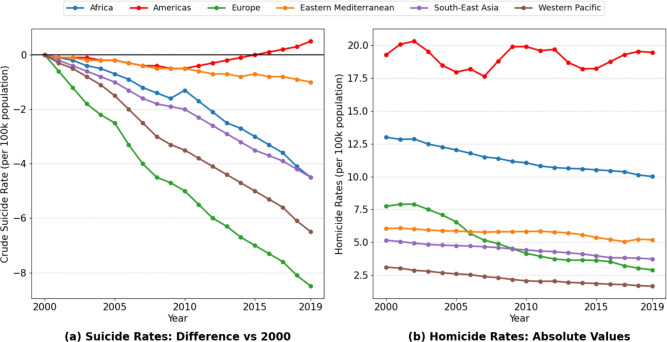
Global yearly trends in homicide and suicide rates by WHO region (2000–2019). (**a**) Suicide rates (per 100,000 population) and (**b**) Homicide rates (per 100,000 population) across six WHO regions: Africa, Americas, Eastern Mediterranean, Europe, South-East Asia, and Western Pacific. Source: WHO Global Health Observatory^1^.

Intentional injury mortality (IIM), encompassing suicides and homicides, constitutes a critical public health challenge with profound social and institutional implications. The Americas, in particular, face a dual crisis, reporting the world’s highest homicide rates and remaining the only region where suicide continues to rise despite considerable economic growth^1,2^. This empirical distinction provides a data-driven rationale for selecting the Americas as the focal region, as it uniquely combines the highest global homicide burden with a persistent upward suicide trajectory. Understanding how governance and macroeconomic dynamics together shape these outcomes is therefore essential for effective prevention and policy design. As illustrated in Fig. 1, Americas is the only region to have illustrated a drastic constant upward suicide trend when compared with the year 2000, while other regions show steady declines (Fig. 1a). Additionally, a recent joinpoint regression analysis confirms that suicide mortality decreased significantly across all World Health Organization (WHO) regions except the Americas, where rates increased in both males and females during 2000–2019 time period^2^. Furthermore, the region consistently exhibit the highest homicide rates worldwide, averaging around 17–20 per 100,000 population between 2000 and 2019 (Fig. 1b).

This pattern underscores the persistence of intentional injuries in the Americas, highlighting the need to examine the structural and institutional mechanisms that sustain these adverse trends. One of the main governance quality measures, corruption defined as the abuse of public office for personal gain, undermines institutional trust, distorts policymaking, and weakens social cohesion^3,4^. In the Americas, corruption has often persisted or worsened, aggravating public frustration when reform expectations are unmet^5–7^. At the same time, macroeconomic pressures such as unemployment, inflation, and unequal growth intensify psychosocial distress and societal unrest^8,9^.These interconnected stressors can manifest itself in both self-directed and interpersonal violence, linking economic instability to collective insecurity. Furthermore, intentional homicide, as defined by the United Nations Office on Drugs and Crime^10^, creates cascading social effects that extend well beyond the immediate victims, undermining the legitimacy of the state and diminishing community trust. Likewise, suicide reflects the buildup of psychological, cultural, and economic stressors^11^, leaving lasting trauma for families and society. As Levi-Belz^12^ highlights, preventing tragedies such as suicide requires sustained, coordinated efforts. However, traditional statistical models often fail to capture the nonlinear and interactive nature of these factors, prompting the use of advanced analytical methods. In response to these analytical limitations, artificial intelligence (AI) and machine learning (ML) techniques have increasingly been recognized as effective tools for modeling complex, multidimensional relationships in social and economic systems. Prior studies highlight their success in capturing nonlinear interactions and interdependent variables in various scenarios and subject arenas where human nature is involved^13–15^. Data-driven early warning systems have also been successfully applied to societal risk monitoring, including the prediction of epidemic outbreaks using large-scale social signals^16^, underscoring the growing role of predictive analytics in anticipating complex public health and social crises. These approaches provide a more flexible and data-driven means of exploring how governance and macroeconomic conditions jointly influence intentional injury mortality.

Despite the magnitude of the problem, region-wide comparative studies of IIM in the Americas remain scarce. Although previous research demonstrates that machine learning techniques can effectively model complex social systems, their application to intentional mortality within a governance and economic framework has been limited. Addressing this gap is essential for developing empirically grounded and policy-relevant models that integrate both immediate and long-term determinants of violence.

Grounded in this rationale, the study focuses on four key predictors: corruption, inflation, unemployment, and economic growth. These variables have been identified across the literature as significant drivers of both homicide and suicide. Table 1 summarizes the main findings of past studies, outlining the positive and negative relationships between these predictors and suicide and homicide outcomes. In this theoritical framework, CORR, HOM, SUI, INFL, UNEMP, and EG denote corruption score, homicide, suicide, annual inflation rate, unemployment rate, and economic growth, respectively. The columns labeled with + and - signs represent positive and negative associations, respectively, as identified in past literature.

**Table 1:** Variables and key supporting studies on suicide and homicide.

Variables	Past studies	
	(+)	(–)
CORR *⟶* SUI	Kularathne et al. (2025)^17^; Sharma et al. (2021)^18^; Yamamura et al. (2012)^19^; Bjørnskov et al. (2010)^20^	
INFL *⟶* SUI	Souresrafil et al. (2025)^21^; Şentürk and Erbay (2022)^22^; Fountoulakis et al. (2014)^23^; Coope et al. (2014)^24^	Lyu et al.(2025)^25^; Botha (2012)^26^; Granados (2008)^27^
UNEMP *⟶* SUI	Obama (2025)^28^; Jung et al. (2024)^29^; Shand et al. (2021)^30^; Milner et al. (2012)^31^	Barth et al. (2011)^32^; Noh (2009)^33^
EG *⟶* SUI	Rajagukguk et al. (2020)^34^; Fountoulakis et al. (2014)^23^	Karasoy (2024)^35^; Mattei and Pistoresi (2019)^36^; Zhang et al. (2010)^37^; Park et al. (2003)^38^
CORR *⟶* HOM	Kularathne et al. (2025)^17^; Croci and Chainey (2023)^39^; ; Chainey et al. (2021)^6^; Poveda et al. (2019)^40^	Ericsson (2024)^7^
INFL *⟶* HOM	Santos et al. (2021)^8^; Rosenfeld and Fox (2019)^41^; Rosenfeld (2014)^42^	
UNEMP *⟶* HOM	Rasvanis (2024)^43^; Schleimer et al.^9^ (2022); Dávila et al. (2019)^44^; Stuckler et al. (2009)^45^	; Ruddell (2017)^46^; South and Cohen (1985)^47^
EG *⟶* HOM	Chainey et al. (2021)^6^; Evans and Topoleski (2002)^48^; Lee (2001)^49^	Enocson (2025)^50^; Gazilas (2024)^51^; Gokmenoglu et al. (2022)^52^; Caraballo Cueto (2015)^53^

As summarized in Table 1, prior studies vary in spatial scope (cross-national, country-level, and subnational), temporal coverage, and reported functional forms. These contextual differences contribute to heterogeneity in the observed associations across the literature.

Despite extensive research examining suicide, homicide, and macroeconomic stressors separately, few studies have integrated governance quality and macroeconomic indicators within a unified, explainable AI framework to model intentional injury mortality at a region-wide scale in the Americas. Moreover, prior work has rarely distinguished between contemporaneous effects and persistence-driven dynamics across income levels and sub-regional contexts. Addressing these gaps, this study provides a temporally structured and interpretable modeling approach that disentangles short-term shocks from long-term structural inertia.

Building on these foundations of prior researchers, the main goal of this study is to examine how governance quality and key macroeconomic indicators, including unemployment, inflation, and economic growth, influence intentional injury mortality across income levels and sub-regional contexts in the Americas. By analyzing these factors collectively through AI-based models, the study seeks to uncover the mechanisms that sustain cycles of violence and self-harm within the region.

This research contributes to the existing literature and policymaking in several essential ways. First, it fills a significant empirical gap by providing one of the most comprehensive region-wide analyses of IIM in the Americas, a region simultaneously facing uncontrollable homicide rates and the world’s only rising suicide trend. Despite this dual burden, systematic comparative analyses that integrate governance and macroeconomic dimensions are limited. Second, the study uses an extensive and contemporary dataset covering the years 2000 to 2019, allowing exploration of long-term patterns and temporal persistence. This approach provides an updated and robust understanding of how structural weaknesses and economic stressors shape violent mortality over time. Third, Explainable artificial intelligence (xAI) is employed as a complementary framework to traditional statistical approaches. While regression-based models are effective for parametric inference under predefined functional forms, machine learning methods allow greater flexibility in capturing non-linear relationships and high-order interactions that may not be easily specified a priori. The integration of SHAP-based explanations enables structured interpretation of complex models by quantifying variable contributions across observations, thereby extending interpretability beyond average marginal effects. This approach enhances the ability to detect heterogeneous and context-specific patterns in macro-structural data while maintaining analytical transparency. In addition, the study adopts a twofold modeling strategy consisting of the snapshot model and the persistence-aware model, which together capture both short-term variations and long-term continuities. The snapshot model identifies the immediate effects of current socioeconomic and governance conditions, while the persistence-aware model uncovers how enduring and self-reinforcing dynamics sustain high levels of intentional injury mortality over time. Finally, the study offers actionable insights for targeted policy interventions. By disaggregating results by income group and subregion, it identifies differentiated priorities. Such insights emphasize that the drivers of IIM are multidimensional and require both immediate and sustained policy responses.

## Methods

### Study design and setting

This study conducted a retrospective, multi-country analysis of intentional injury mortality across the Americas, using annual national-level datasets from 2000 to 2019, spanning two decades, gathered from publicly available international sources. The period from 2000 to 2019 was selected to ensure temporal consistency across all data sources and to capture two decades of regional socioeconomic and health dynamics preceding the COVID-19 pandemic. Table 2 provides the variable codes, measurement units, and data sources used in this study.

**Table 2:** Data variable definitions and sources.

Variable code	Variable	Measurement unit	Source
CORR	Corruption score	Corruption perception index (0–100)	Transparency International, Corruption Perceptions Index (CPI)
HOM	Homicides	Homicide rate (per 100,000 population)	World Health Organization (WHO), Global Health Observatory
SUI	Suicides	Crude suicide rate (per 100,000 population)	World Health Organization (WHO), Global Health Observatory
INFL	Inflation	Inflation, consumer prices (annual %)	The World Bank, World Development Indicators
UNEMP	Unemployment	Unemployment rate, total (% of total labour force), modelled ILO estimate	The World Bank, World Development Indicators
EG	Economic growth	GDP growth (annual %)	The World Bank, World Development Indicators

Source: Authors’ compilation based on data from the World Bank^54^, Transparency International^55^, and the World Health Organization^56^.

As this study used only publicly available, reliable secondary data, it did not involve human subjects directly. Therefore, formal ethics approval was not required in accordance with institutional and international research ethics guidelines.

The variables listed below were used in the analysis.

- The Corruption Perceptions Index (CPI) measures perceived levels of public sector corruption across countries, assigning a score from 0 (highly corrupt) to 100 (very clean)^55^.
- Homicides (HOM) and Suicides (SUI) represent intentional injury mortality components. According to the WHO, suicide is defined as the deliberate act of taking one’s own life, while homicide is defined as the intentional killing of another person, excluding deaths from lawful interventions or military operations^1,56^.
- Inflation (INFL) is the annual percentage change in consumer prices based on the Laspeyres formula^57^.
- Unemployment (UNEMP) is the percentage of the total labour force that is unemployed but actively seeking work^58^.
- Economic growth (EG) represents the annual percentage growth rate of GDP at market prices based on constant 2015 US dollars, reflecting the total value added by all resident producers plus product taxes and minus subsidies^59^.
- The primary outcome, intentional injury mortality (IIM), was operationalized as the sum of the crude suicide rate and the homicide rate per 100,000 population for each country-year.

In this study, intentional injury mortality (IIM) refers exclusively to the suicide–homicide composite; conflict-related deaths and legal executions are not included due to definitional heterogeneity and cross-country comparability considerations. All variables were measured at the national level and aligned by calendar year, resulting in an unbalanced country-year panel comprising up to 25 countries over the 2000–2019 period.

### Data sources and variables

#### Overview of datasets

**Fig. 2:**
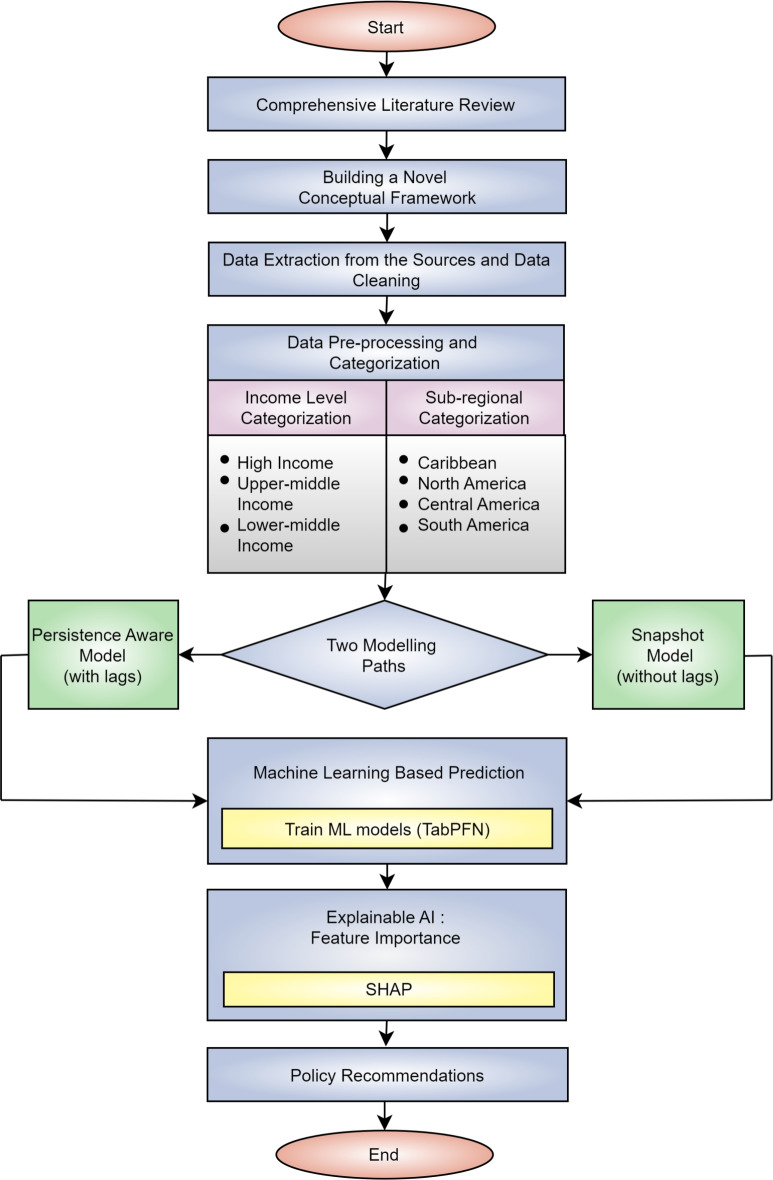
Workflow of the study showing the overall methodological framework.

Figure 2 illustrates the methodological workflow of this study.

This study employed a twenty year panel dataset (2000–2019) from 25 American countries to analyze the structural and temporal factors influencing intentional injury mortality (IIM). Two predictive modelling approaches were developed: a snapshot model using contemporaneous macro-social indicators, and a persistence-aware model incorporating both lagged IIM data and lagged predictors to account for temporal dependence. The independent factors included corruption perception, unemployment, inflation, and GDP growth, which are common indicators of socio-economic stress and governance quality. The analytical unit was the country–year, yielding up to approximately 500 observations across the study period.

#### Country selection, income and sub-regional grouping

Grouping by both income level and subregion allows the models to capture heterogeneity associated with socioeconomic development and geographic context, improving interpretability of region-specific drivers of intentional injury mortality. Countries were classified by income level using the World Bank’s 2024 income categorization and by geographic subregion following the United Nations Statistics Division (UNSD) M49 geoscheme. In the income-level categorization, high-income (n = 8), upper-middle income (n = 13), and lower-middle income (n = 4) countries were included. The study excludes low-income countries because, according to the World Bank, none exist in the American region^60^. The sub-regional categorization includes Caribbean (n = 5), Central America (n = 7), North America (n = 2), and South America (n = 11)^61^. The countries included in each category are presented in Table 3.

**Table 3:** Income-level and sub-regional categorization of countries included in the analysis.

Income-level categorization			Sub-regional categorization			
High income	Upper-middle income	Lower-middle income	Caribbean	Central America	North America	South America
Barbados Canada Chile Guyana Panama Trinidad and Tobago USA Uruguay	Argentina Brazil Colombia Costa Rica Dominican Republic Ecuador El Salvador Guatemala Jamaica Mexico Paraguay Peru Suriname	Bolivia Haiti Honduras Nicaragua	Barbados Dominican Republic Haiti Jamaica Trinidad and Tobago	Costa Rica El Salvador Guatemala Honduras Mexico Nicaragua Panama	Canada USA	Argentina Bolivia Brazil Chile Colombia Ecuador Guyana Paraguay Peru Suriname Uruguay

To clarify the implementation, the income-level and sub-regional analyses were conducted through stratified model estimation rather than by introducing income or regional indicators as categorical variables within a single pooled model. The country-year panel was partitioned into sub-samples according to the World Bank income classifications and United Nations sub-regional groupings, and separate models were trained and evaluated within each subgroup. The unit of analysis remains the country–year observation in all specifications. This stratified approach allows structural heterogeneity in predictor importance and temporal dynamics to be examined explicitly across different socioeconomic and geographic contexts.

Data availability varied slightly across indicators; country-years with missing values in any variable were handled as described in the following subsection on data preprocessing.

#### Data preprocessing, missing values and outlier analysis

Prior to model development, all datasets were harmonized by aligning variable names, measurement units, and country codes across data sources. Annual records were merged into a unified country-year panel covering the 2000–2019 period.

Missing values in predictor variables (*CORR*, *INFL*, *UNEMP*, and *EG*) and outcome variables (*HOM*, *SUI*, and *IIM*) were infrequent and limited to isolated years. To maintain temporal continuity and avoid unnecessary data loss, missing observations were imputed using the mean of the immediately preceding and succeeding years within each country. When one or both adjacent observations were unavailable (e.g., at the beginning or end of a time series), the country-specific mean of the respective variable was used. This approach preserved longitudinal structure while minimizing distortion of short-term trends.

All variables were retained in their original measurement scales. No logarithmic transformation or standard-score normalization was applied, as the tree-based and transformer-based architectures employed in this study are invariant to monotonic transformations and do not require feature scaling for stable performance.

Outlier detection was conducted prior to model training through a multi-stage inspection process, including visual screening of distribution plots and evaluation of summary statistics. Extreme observations were assessed relative to the theoretical and historically plausible bounds of each socioeconomic indicator. No implausible or data-entry anomalies were identified. Although formal influence diagnostics such as Cook’s Distance are primarily designed for parametric linear models, the selected tree-based and transformer-based frameworks are inherently less sensitive to extreme feature values. Consequently, all verified observations were retained to preserve the full range of regional variability and temporal volatility within the panel.

The resulting dataset constitutes a consistent and complete country-year panel suitable for predictive modeling and explainability analysis.

### Modeling framework

Two complementary modeling paradigms were developed to estimate and interpret the dynamics of intentional injury mortality across the Americas: a *snapshot model* and a *persistence-aware model*. The snapshot model relied solely on contemporaneous socio-economic indicators to capture cross-sectional structural associations. This lagged Vs non-lagged comparison is recommended to reveal immediate Vs history-influenced effects on economic development issues^62^. In contrast, the persistence-aware model extended this framework by incorporating temporal memory through lagged predictors and lagged outcome values, thereby accounting for persistence effects and temporal autocorrelation.

#### Model families and selection

A total of 28 state-of-the-art (SOTA) regression algorithms were systematically evaluated to identify the best-performing framework for IIM prediction. These models encompassed six broad families:

1. Linear and generalized linear models (e.g., Linear, Robust Linear, Stepwise Linear, Interactions Linear);
2. Decision-tree ensembles (e.g., Fine, Medium, and Coarse Trees, Boosted Trees, Bagged Trees);
3. Support Vector Machines (Linear, Quadratic, Cubic, and Gaussian kernels of varying scales, and Efficient Linear SVM);
4. Gaussian Process Regressors (Squared Exponential, Matern 5/2, Exponential, and Rational Quadratic kernels);
5. Artificial Neural Networks (Narrow, Medium, Wide, Bilayered, and Trilayered networks);
6. Kernel and Least-Squares variants (SVM Kernel, Efficient Linear Least Squares, Least Squares Regression Kernel).

All benchmark regression models and the TabPFN framework were trained on identical input–output mappings using mean-squared error (MSE), or the equivalent squared-error regression objective where implemented by the underlying library, as the primary optimization criterion to ensure comparability across model families. Model optimization followed the generic cost function:

1 $$\begin{aligned} J(\theta ) = \frac{1}{N}\sum _{i=1}^{N}\left( y_i - \hat{y}_i(\theta )\right) ^2, \end{aligned}$$

where $y_i$ denotes the observed IIM rate for country–year *i*, $\hat{y}_i(\theta )$ the model-predicted value parameterized by *θ*, and *N* the total number of training observations. The objective was to minimize *J(θ )* through iterative parameter estimation appropriate to each algorithmic class (e.g., gradient boosting for trees, back-propagation for neural networks, and quadratic optimization for SVMs).

### Model selection and performance metrics

To identify the most robust framework for predicting intentional injury mortality, we systematically evaluated 28 state-of-the-art regression algorithms encompassing six broad families: linear models, tree ensembles, support vector machines, Gaussian process regressors, and neural networks. Model performance was assessed using five complementary metrics: mean squared error (MSE), root mean squared error (RMSE), coefficient of determination ($R^2$), mean absolute error (MAE), and mean absolute percentage error (MAPE).

Across all experiments, the *TabPFN* model exhibited the most consistent generalization performance and interpretability, outperforming other models across income and sub-regional groups. Consequently, TabPFN was adopted as the final model for both the snapshot and persistence-aware frameworks. Full comparative results and hyperparameter configurations for all tested algorithms are provided in S1 Supplementary Document (Table S1 and S2) while the top 10 model results are summarized in Table 4. For transparency, Table S1 reports the full comparative performance of all evaluated models, including TabPFN, across MSE, RMSE, $\textrm{R}^{2}$, MAE, and MAPE.

**Table 4:** Comparative performance analysis of evaluated ML models (test set results).

ML model	Test RMSE	Test $R^2$	Test MAE
TabPFN	1.76	0.98	1.46
Narrow neural network	5.16	0.92	4.00
Cubic SVM	5.65	0.90	4.12
Medium neural network	5.70	0.90	4.15
Matern 5/2 GPR	5.92	0.89	4.45
Squared exponential GPR	5.94	0.89	4.44
Rational quadratic GPR	5.97	0.89	4.51
Exponential GPR	6.18	0.88	4.69
Quadratic SVM	6.30	0.87	5.38
Medium Gaussian SVM	6.34	0.87	5.30

#### Snapshot and persistence-aware models

In the *snapshot model*, predictors included current-year values of corruption perception ($\textit{CORR}_{t}$), inflation ($\textit{INFL}_{t}$), unemployment ($\textit{UNEMP}_{t}$), and economic growth ($\textit{EG}_{t}$) to estimate contemporaneous IIM rates ($\textit{IIM}_{t}$).

The *persistence-aware model* augmented this structure by incorporating lagged variables to capture temporal dependencies. For each predictor and the outcome variable, lags up to five years (*t-1* to *t-5*) were examined, and the most informative lag combination was selected empirically based on validation performance and feature-importance diagnostics. The resulting model thus captured both immediate and delayed socio-economic effects on IIM.

The distinction between the snapshot and persistence-aware frameworks reflects differences in temporal structure rather than variable selection. The predictors included in the models were not selected through exploratory screening but were theoretically grounded drivers identified in prior literature. While variable selection techniques such as VIF or LASSO can address multicollinearity or sparsity within a single specification, they do not substitute for modeling temporal dependence. Because intentional injury mortality exhibits temporal autocorrelation, combining contemporaneous and lagged variables within a single selection-based framework may obscure the interpretability of immediate versus history-dependent effects. The use of two separate specifications therefore allows explicit evaluation of short-term structural sensitivity and longer-term persistence, consistent with established lag-structure approaches in social science research.

#### TabPFN model and hyperparameters

TabPFN is a transformer-based meta-learning architecture designed for small to medium-sized tabular datasets. It infers Bayesian posterior predictions by implicitly learning a prior over functions from millions of synthetic training tasks. The principal hyperparameters are summarized in Table 5.

**Table 5:** Default hyperparameters used for the TabPFN model.

Parameter	Value / description
Model type	TabPFN transformer (pre-trained)
Input dimension	Number of selected predictors per model
Number of transformer layers	12
Hidden size	512
Number of attention heads	8
Dropout rate	0.1
Learning rate	1e-4 (Adam optimizer)
Batch size	128
Loss function	Mean squared error (MSE)
Epochs (effective inference passes)	1 (single forward inference)

All TabPFN models were implemented in Python 3.11 using the official tabpfn library. Hyperparameter optimization for the baseline algorithms, along with further ablation results, are documented in S1 Supplementary (Table S1 and S2).

### Model training and validation

Model training and performance evaluation were carried out using a consistent experimental design across all tested algorithms, including the TabPFN and the 28 state-of-the-art baseline models.

To ensure robust generalization and avoid temporal leakage, a five-fold cross-validation scheme was employed for each income level and sub-regional model configuration. In this approach, the full 2000–2019 panel was partitioned into five temporally contiguous folds; each fold served once as the validation set. This time-aware design prevented information from future years from leaking into model training and allowed unbiased estimation of predictive accuracy. Moreover, the purpose of using k-fold cross-validation in this study was to ensure that the model performs reliably across different data segments, further demonstrating its robustness^63^.

Model performance was assessed using five complementary evaluation metrics: mean squared error (MSE), root mean squared error (RMSE), coefficient of determination ($R^2$), mean absolute error (MAE), and mean absolute percentage error (MAPE). These measures collectively quantify predictive precision, variance explanation, and proportional error. The corresponding formulations are given below.

2 $$\begin{aligned} \textrm{MSE} = \frac{1}{N} \sum _{i=1}^{N} \left( y_i - \hat{y}_i \right) ^2, \end{aligned}$$

where $y_i$ and $\hat{y}_i$ denote the observed and predicted IIM rates for observation *i*, respectively, and *N* is the number of observations. MSE penalizes large errors more heavily and was used as the primary optimization objective for model training^64^.

3 $$\begin{aligned} \textrm{RMSE} = \sqrt{ \frac{1}{N} \sum _{i=1}^{N} \left( y_i - \hat{y}_i \right) ^2 }, \end{aligned}$$

which expresses the error in the same units as the outcome variable, facilitating direct interpretability of model deviations^65^.

4 $$\begin{aligned} R^2 = 1 - \frac{\sum _{i=1}^{N} (y_i - \hat{y}_i)^2}{\sum _{i=1}^{N} (y_i - \bar{y})^2}, \end{aligned}$$

where $\bar{y}$ is the sample mean of observed IIM values. The $R^2$ statistic measures the proportion of total variance in the observed data explained by the model^66^.

5 $$\begin{aligned} \textrm{MAE} = \frac{1}{N} \sum _{i=1}^{N} \left| y_i - \hat{y}_i \right| , \end{aligned}$$

which represents the average magnitude of prediction errors regardless of direction and provides a linear measure of model accuracy^67^.

6 $$\begin{aligned} \textrm{MAPE} = \frac{100}{N} \sum _{i=1}^{N} \left| \frac{y_i - \hat{y}_i}{y_i} \right| , \end{aligned}$$

which quantifies average relative error as a percentage of observed values, enabling comparison across models and scales^68^. MAPE was used as a complementary indicator to assess proportional deviations, particularly for cross-country comparisons where baseline IIM rates differ.

To prevent temporal data leakage, model training and evaluation were conducted using a strictly time-ordered split. Observations from earlier years were used for model training, while subsequent years were reserved exclusively for testing. Lagged variables were constructed within each temporal window to ensure that no information from future periods entered the training set. This chronological separation preserves the predictive integrity of the evaluation framework.

All metrics were computed for each fold and then averaged to yield the final performance estimates reported in the Results section. Complete cross-validation outcomes and model-wise metric comparisons are provided in S1 Supplementary Document (Table 1).

### Explainable AI / SHAP analysis

To interpret the contribution of each explanatory variable toward predicted IIM values, the study employed SHapley Additive Explanations (SHAP), a model-agnostic framework grounded in cooperative game theory^69^. SHAP decomposes each model prediction into additive feature attributions, quantifying how much each variable increases or decreases the predicted outcome relative to the global mean prediction.

For the tree-based baseline models, SHAP values were computed using the TreeExplainer algorithm. In contrast, for the transformer-based TabPFN model, feature attributions were derived using KernelExplainer, a model-agnostic method. SHAP values ($\phi _j$) for each feature *j* were obtained as follows:

7 $$\begin{aligned} \phi _j = \sum _{S \subseteq F \setminus \{j\}} \frac{|S|!\,(|F| - |S| - 1)!}{|F|!} \left[ f_{S \cup \{j\}}(x_{S \cup \{j\}}) - f_S(x_S) \right] , \end{aligned}$$

where *F* denotes the full set of features, *S* represents any subset of features excluding *j*, and $f_S(x_S)$ is the model prediction using only features in *S*. The term in brackets measures the marginal contribution of feature *j* to the prediction, and the weighting term ensures fair attribution across all possible feature coalitions. The model prediction for an observation *i* can therefore be expressed as:

8 $$\begin{aligned} \hat{y}_i = \phi _0 + \sum _{j=1}^{M} \phi _{ij}, \end{aligned}$$

where $\phi _0$ is the expected prediction across all observations and $\phi _{ij}$ denotes the SHAP contribution of feature *j* for sample *i*.

To ensure interpretive validity, SHAP values were computed from out-of-sample predictions obtained during cross-validation rather than training data, thereby preventing information leakage and over-attribution. The direction and magnitude of each feature’s influence were visualized using summary beeswarm plots, dependence plots, and mean absolute SHAP rankings within each income level and sub-regional subgroup.

Feature-level interpretations were subsequently aggregated to identify dominant structural and temporal drivers of IIM across the Americas. This explainability analysis enabled transparent understanding of the persistence-aware and snapshot models, allowing comparison of variable influence patterns across differing socio-economic contexts.

## Results

### Descriptive temporal analysis

**Fig. 3:**
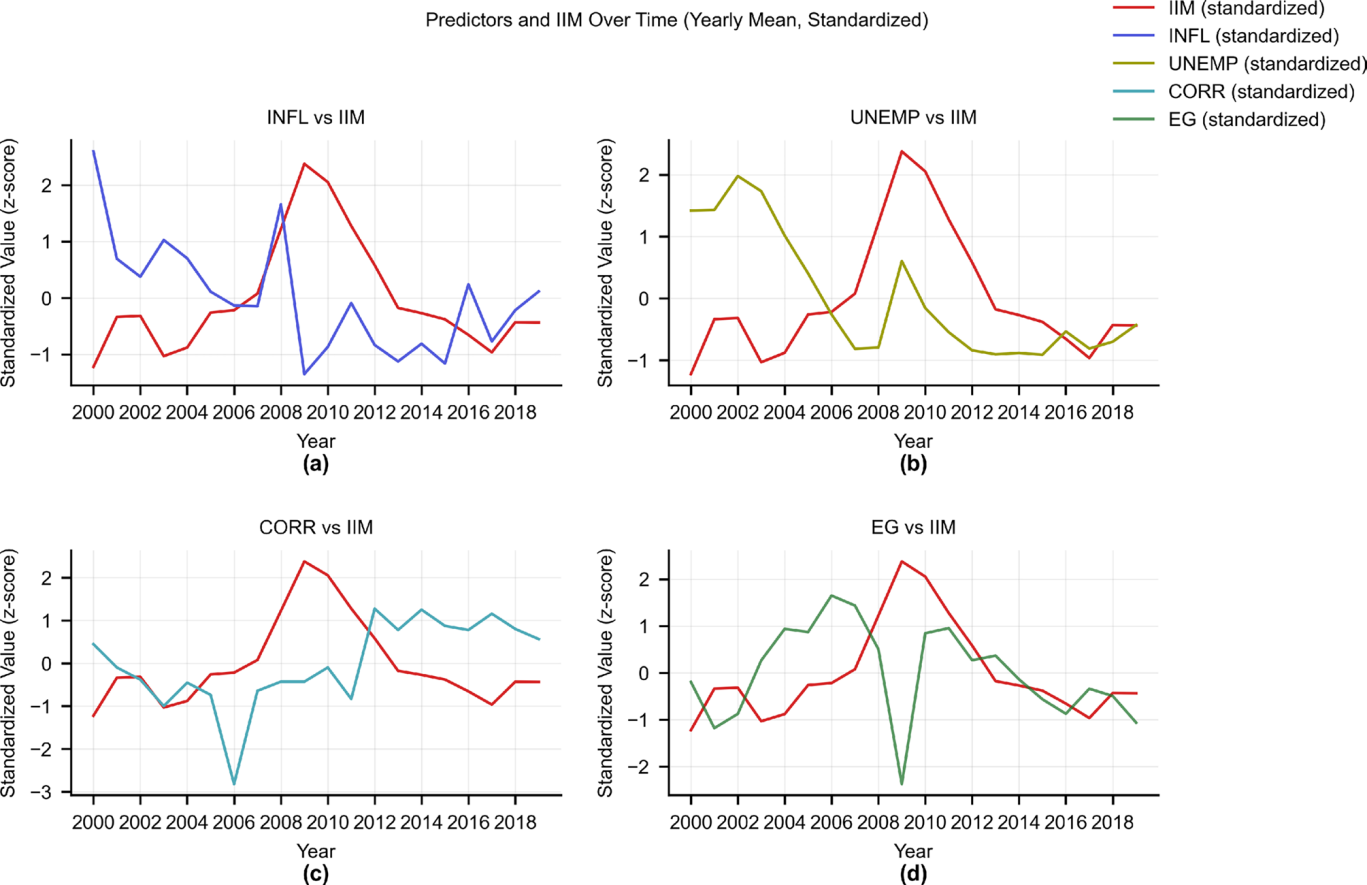
Temporal trajectories of intentional injury mortality and key predictors in the Americas (2000–2019). Panels display yearly regional mean values (standardized as z-scores) for intentional injury mortality (IIM) and each predictor: (**a**) Inflation (INFL) vs. IIM, (**b**) unemployment (UNEMP) vs. IIM, (**c**) corruption perception (CORR) vs. IIM, and (**d**) economic growth (EG) vs. IIM. Standardization allows comparison of relative fluctuations over time across variables with different original units.

Figure 3 presents the temporal trajectories of intentional injury mortality (IIM) and key predictors based on yearly regional means (standardized) from 2000 to 2019.

IIM shows a marked increase during the late 2000s, followed by gradual stabilization in the subsequent decade. Inflation and unemployment exhibit cyclical fluctuations, particularly around the global financial crisis period, partially coinciding with shifts in IIM. Economic growth displays pronounced volatility during crisis years, while corruption trends show more gradual structural variation over time. Overall, the descriptive time-series patterns indicate that macroeconomic instability and governance dynamics evolve alongside changes in intentional injury mortality, supporting the inclusion of both contemporaneous and lagged predictors in the modeling framework. These temporal patterns provide preliminary descriptive evidence of co-movement and volatility among the predictors and IIM, motivating the subsequent modeling analysis to formally quantify both contemporaneous and persistence-driven relationships.

### Income-stratified dynamics of intentional injury mortality

**Fig. 4:**
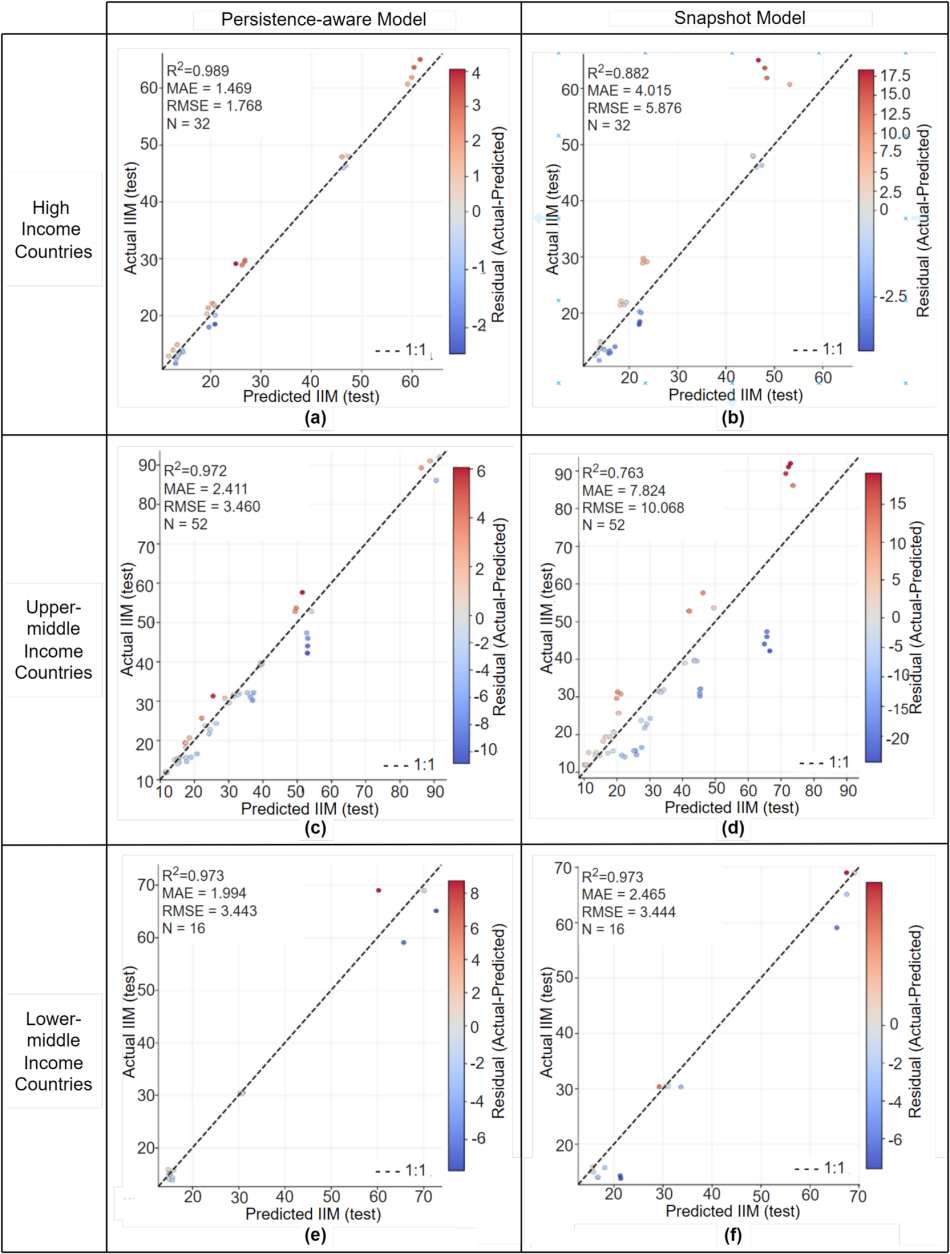
Income-stratified predictive performance of persistence-aware and snapshot models. Test-set predictions versus observed intentional injury mortality (IIM) are shown for high-, upper-middle-, and lower-middle-income countries under both persistence-aware and snapshot model specifications. Each panel reports model fit statistics ($R^{2}$, MAE, RMSE, and sample size *N*), with point colours indicating residuals (actual minus predicted). Intentional injury mortality (IIM) is defined as the sum of crude suicide and homicide rates per 100,000 population, and predicted values are expressed in the same units.

Figure 4 compares the predictive accuracy of the snapshot and persistence-aware models across World Bank income groups. The persistence-aware formulation consistently attains higher coefficients of determination ($\textrm{R}^{2}$ = 0.98–0.97 versus 0.97–0.76 for the snapshot) and lower mean absolute error (MAE = 1.47–2.41 versus 2.46–7.82), with residuals tightly clustered around the $45^{\circ }$ line. The improvement is most pronounced among upper-middle- and high-income economies, where year-to-year volatility is higher. This pattern confirms that incorporating prior-year IIM captures structural inertia, reflecting social, psychological, and institutional legacies that contemporaneous socioeconomic indicators alone cannot represent. The snapshot model, by contrast, remains helpful in exposing short-run fluctuations that show how immediate shocks in corruption, unemployment, or inflation correspond to short-term IIM changes. The persistence-aware model extends this view by revealing the embedded continuity of violence and self-harm, illustrating how social and institutional damage persists once triggered.

**Fig. 5:**
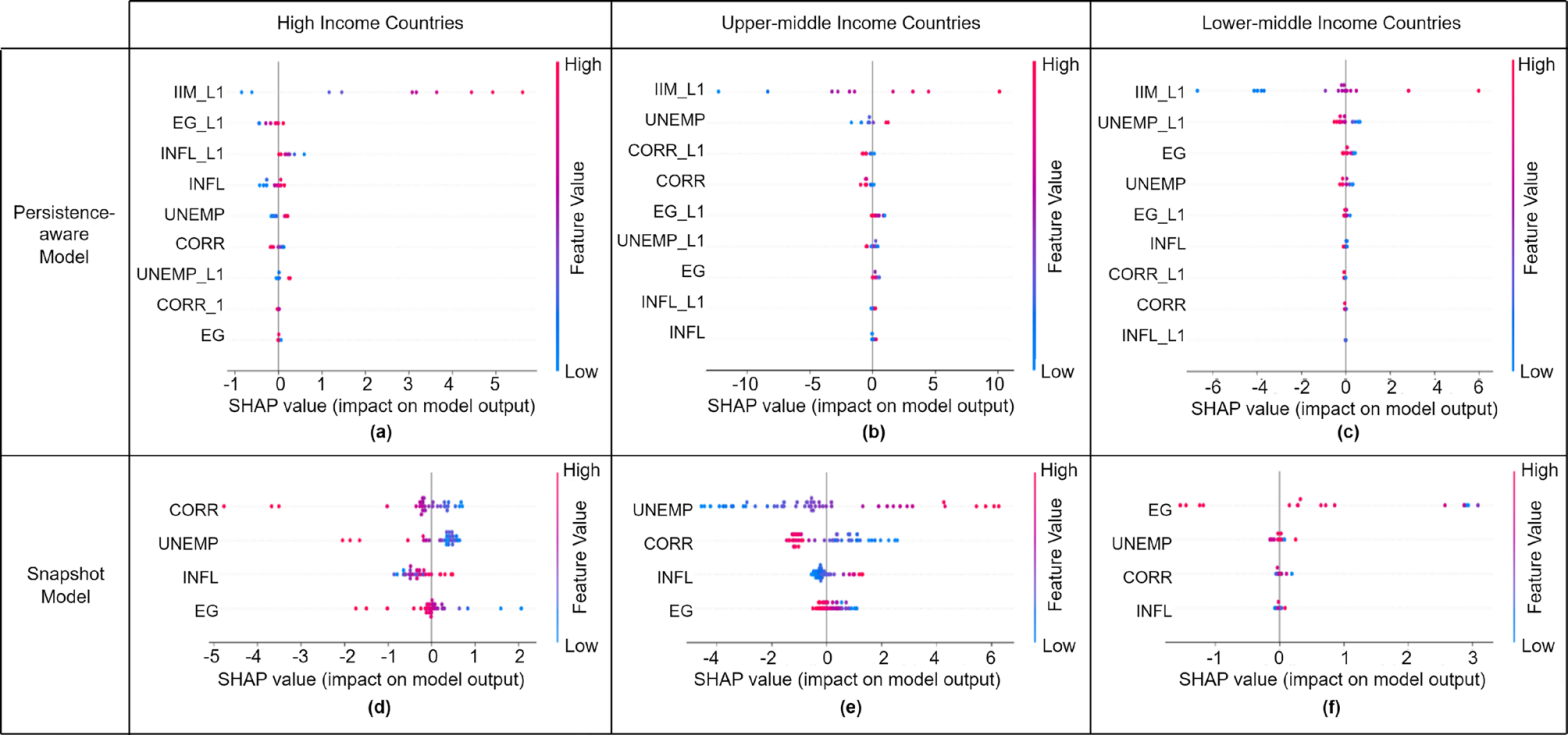
Income-stratified SHAP impacts for snapshot and persistence-aware models. Panels show the directional influence of each predictor on intentional injury mortality across high-, upper-middle-, and lower-middle-income countries. IIM, CORR, UNEMP, INFL, and EG denote intentional injury mortality, perceived corruption, unemployment rate, annual inflation, and economic growth, respectively. Predictors with the suffix L1 correspond to their one-year lagged values, reflecting the influence of prior-year socioeconomic and mortality conditions on current outcomes. SHAP values indicate the direction and magnitude of each predictor’s contribution to the predicted IIM (per 100,000 population)..

Figure 5 illustrates SHAP (SHapley Additive Explanations) distributions, highlighting each feature’s directional influence for theincome levels of the Americas under two modeling frameworks: the persistence-aware model and the snapshot model. The given SHAP explanation is represented by a single dot on each feature row for each sample. The SHAP value of each feature determines the x position of the dot, and dots pile up along each feature row to show density. Color indicates the original value of the feature.

In high-income countries, corruption shows the steepest positive SHAP gradients in the snapshot model, indicating that governance decline quickly raises IIM. When persistence is included, corruption’s effect becomes more stable and is partly absorbed by the lagged IIM term, suggesting that institutional trust deficits build up over time rather than appearing as isolated shocks. In upper-middle-income countries, unemployment dominates both models, although the persistence-aware version reveals that much of its effect is transmitted through previously high IIM. This suggests that job losses are most harmful in already fragile social systems. In lower-middle-income economies, GDP growth consistently acts as a safeguard: higher growth lowers predicted IIM, and this relationship becomes stronger after accounting for persistence.

**Fig. 6:**
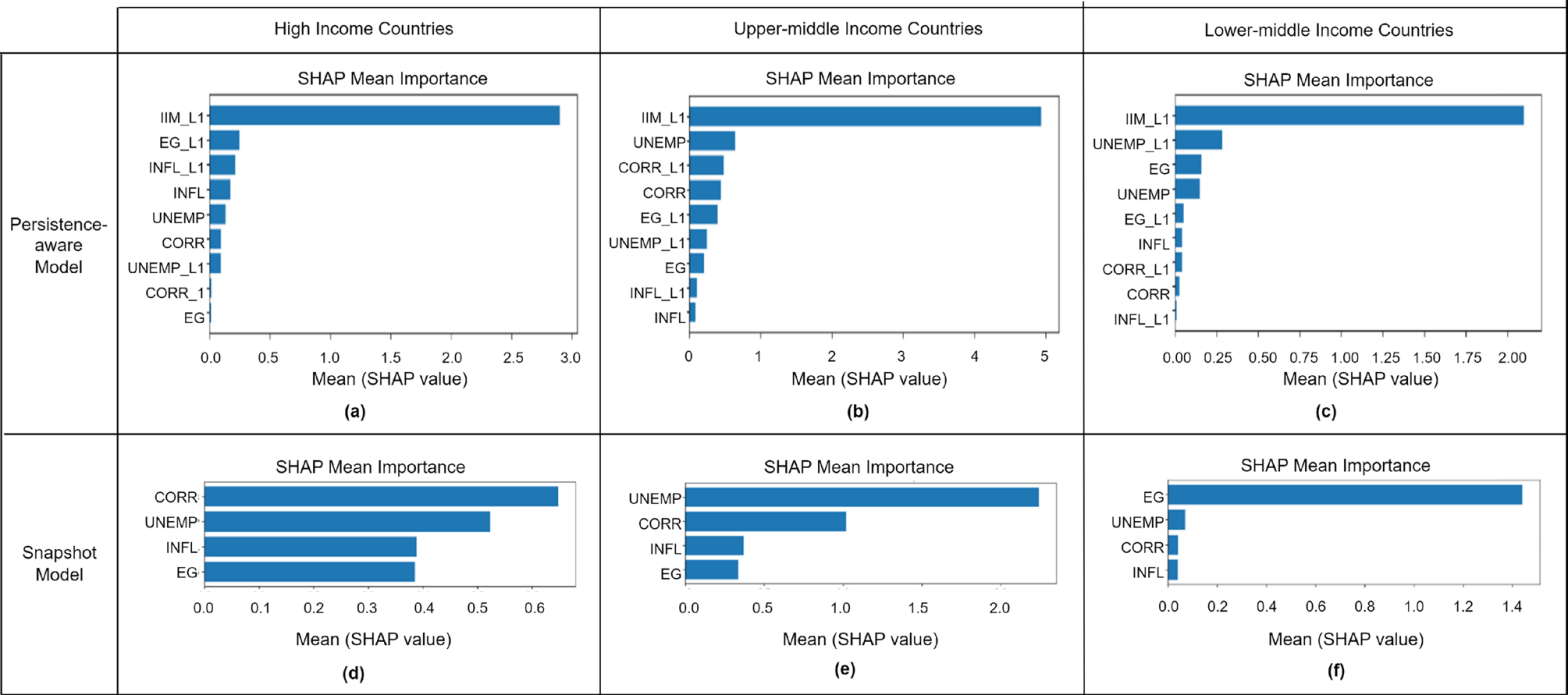
Income-stratified SHAP mean importance for persistence-aware and snapshot models. Mean SHAP values highlight the relative influence of socioeconomic and governance factors on intentional injury mortality across high-, upper-middle-, and lower-middle-income countries. IIM, CORR, UNEMP, INFL, and EG denote intentional injury mortality, perceived corruption, unemployment rate, annual inflation, and economic growth, respectively, while variables with the suffix L1 represent their one-year lagged values. SHAP values represent contributions to the model’s predicted IIM (per 100,000 population), and mean absolute SHAP values reflect average contribution magnitude across observations..

Figure 6 ranks variables by mean absolute SHAP value and reveals an interesting divergence in the leading contemporaneous drivers across income groups. In high-income countries, corruption remains the most influential factor, aligning with the idea that institutional decay and governance fatigue amplify both homicide and suicide risk. Among upper-middle-income economies, unemployment is dominant, underscoring how labor-market instability directly undermines social cohesion and psychological well-being. For lower-middle-income countries, GDP growth exerts the most significant protective influence, showing that sustained economic expansion can buffer social stress and reduce vulnerability. In low-income contexts, inflation stands out as the most sensitive contemporaneous stressor, where short-term price surges rapidly translate into welfare erosion and increased mental distress. These variations underscore that each development tier faces distinct immediate risks, even while sharing long-term mortality persistence, highlighting the importance of tailored policy interventions that address both current pressures and enduring structural weaknesses.

Overall, the income-stratified evidence supports three conclusions. First, IIM exhibits strong temporal persistence: past mortality levels remain the single most powerful predictor of future risk. Second, the structure of contemporaneous determinants differs systematically with national income, revealing a meaningful economic gradient in both type and direction of influence. Third, the persistence-aware approach not only enhances fit and reduces error but also refines interpretability. Once history is acknowledged, effect sizes stabilize and relative importance rankings become more coherent for policy design. The contrast between models therefore underscores the dual nature of IIM: short-run socioeconomic sensitivity nested within long-run institutional memory. In general, to isolate the contemporaneous net contribution of socioeconomic variables independent of temporal persistence, the snapshot model results provide a direct comparison, as this specification excludes lagged IIM and lagged predictors.

To ensure the findings are not dependent on a specific algorithmic architecture, a robustness check was conducted comparing the primary TabPFN model against a Medium Gaussian Support Vector Machine (SVM). As detailed in the S1 Supplementary Document (Table 3), the SHAP-based feature importance rankings remained consistent across both models for all three income levels. The Medium Gaussian SVM successfully replicated the importance trends identified by TabPFN, specifically confirming the dominance of unemployment in mid-income tiers and economic growth in low-income tiers. This cross-model validation underscores the robustness of the identified drivers and mitigates concerns regarding model-specific overfitting.

Multicollinearity diagnostics including the lagged dependent variable were conducted and are reported in the S2 Supplementary Document. All variance inflation factor values were low (maximum = 1.24), indicating negligible collinearity among structural predictors and the lagged term.

### Sub-regional dynamics of intentional injury mortality

**Fig. 7:**
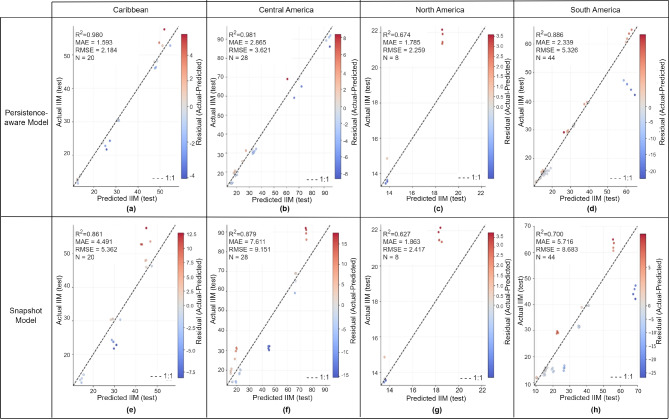
Regional predictive performance of persistence-aware and snapshot models. Scatterplots show test-set predictions versus observed intentional injury mortality (IIM) for Caribbean, Central American, North American, and South American regions. Each panel reports model fit statistics including $R^{2}$, MAE, RMSE, and sample size (*N*), with point colours representing residuals (actual minus predicted). Intentional injury mortality (IIM) is defined as the sum of crude suicide and homicide rates per 100,000 population, and predicted values are expressed in the same units.

Figure 7 compares model performance across the Caribbean, Central America, North America, and South America for both persistence-aware and snapshot models. The persistence-aware framework clearly improves predictive accuracy, with $\textrm{R}^{2}$ values ranging from 0.88 to 0.98, except in North America, where the difference in model fit was comparatively less significant, and the persistence-aware model still had higher predictive accuracy than the snapshot model (0.67 vs 0.62). $\textrm{R}^{2}$ values in all other sub-regions ranged from 0.70 to 0.88, indicating acceptable accuracy but lower than that of the persistence-aware model, suggesting that the persistent nature of the sub-regional models is more prominent than contemporaneous effects in this study. Correspondingly, MAE and RMSE values decrease significantly in the persistence-aware model, indicating minor residual errors and greater model stability.

**Fig. 8:**
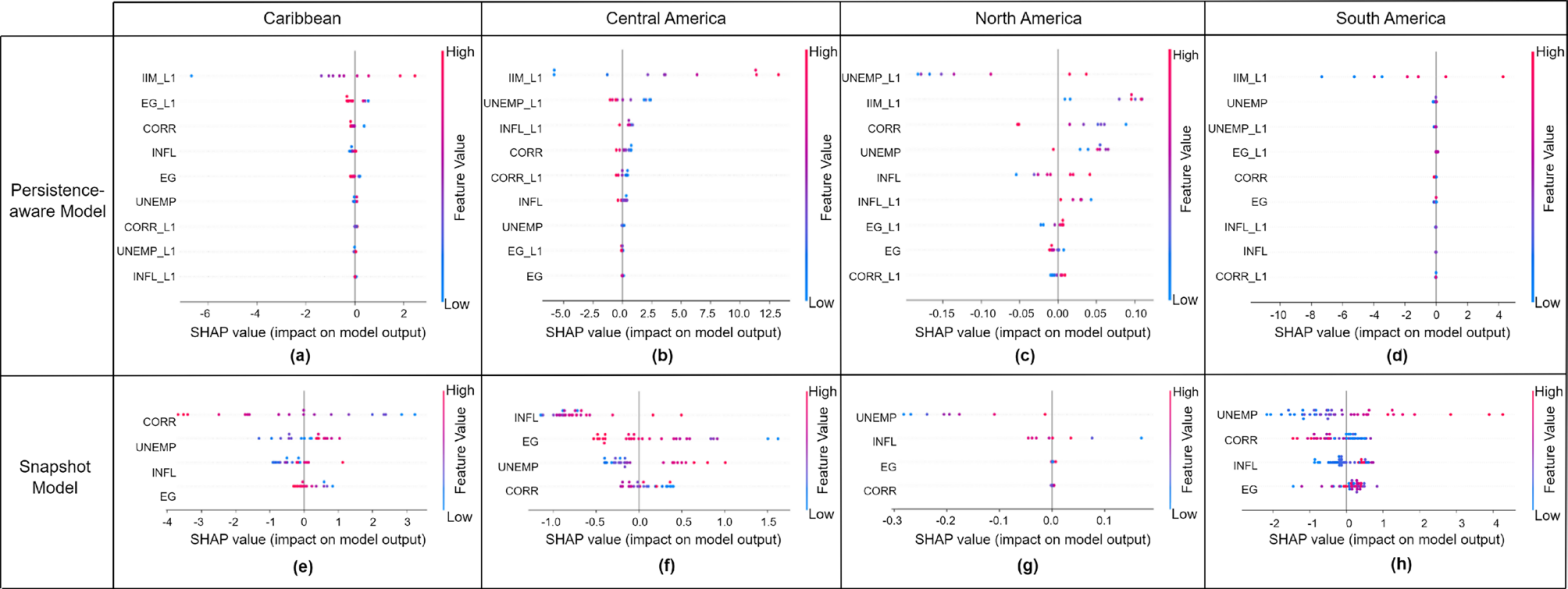
Regional SHAP impacts for persistence-aware and snapshot models. Panels show the directional influence of key predictors on intentional injury mortality (IIM) across Caribbean, Central American, North American, and South American regions. IIM, CORR, UNEMP, INFL, and EG denote intentional injury mortality, perceived corruption, unemployment rate, annual inflation, and economic growth, respectively. Predictors with the suffix L1 indicate their one-year lagged values, capturing delayed effects of prior-year socioeconomic and mortality conditions on current outcomes. SHAP values indicate the direction and magnitude of each predictor’s contribution to the predicted IIM (per 100,000 population)..

Figure 8 presents beeswarm summary plots of representative SHAP feature contributions for the sub-regions of the Americas under two modeling frameworks: the persistence-aware model and the snapshot model, explaining both history-dependent as well as contemporaneous influence on the model output.

The persistence-aware model captures temporal dependence by integrating lagged predictors, revealing that prior-year intentional injury mortality is the strongest positive contributor to current IIM across all sub-regions except North America. In North America, lagged unemployment surpasses lagged IIM, indicating that historical labor-market instability outweighs mortality persistence. In the Caribbean, while lagged IIM remains dominant, lagged EG, EG, and CORR negatively affect IIM, whereas INFL shows a positive contribution, suggesting that rising prices increase mortality risk. In Central America, lagged UNEMP, lagged INFL, CORR, and lagged CORR rank among the most influential factors after lagged IIM. North America again diverges, with lagged IIM ranking second and labor-market instability exerting the strongest effect, while lagged INFL and corruption variables show negative contributions. South America exhibits a wider SHAP range for lagged IIM (–10 to 4), highlighting strong persistence alongside unemployment effects. Lagged unemployment shows modest positive interactions, indicating that increased labor-market stress elevates intentional injury mortality.

The snapshot model, in contrast, emphasizes the immediate influences of negatively affecting CORR in the Caribbean, Central America, and South America, indicating that a rise in Corruption Score (decrease in corruption levels) can increase IIM rates. The rise in UNEMP rates has a positive influence across all sub-regions. At the same time, the EG visualizes a clearly strong negative impact on the model output in the Caribbean and Central America. South America had a greater positive impact, and North America did not significantly influence the Snapshot model’s output, highlighting that while short-term shocks explain contemporaneous fluctuations, persistence-aware modeling captures deeper structural and historical drivers of IIM across the Americas.

**Fig. 9:**
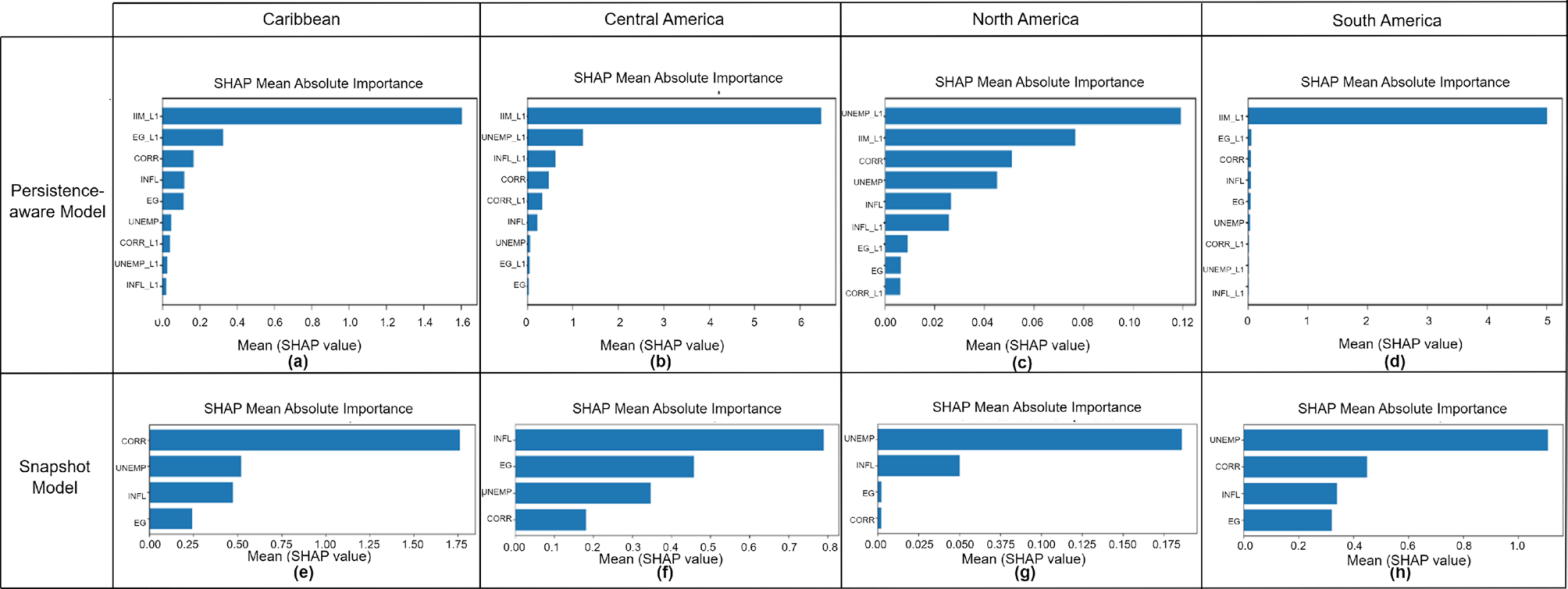
Regional SHAP mean absolute importance: persistence-aware vs. snapshot models. Mean absolute SHAP values summarize the relative importance of each predictor for explaining intentional injury mortality (IIM) across Caribbean, Central American, North American, and South American regions. IIM, CORR, UNEMP, INFL, and EG represent intentional injury mortality, perceived corruption, unemployment rate, annual inflation, and economic growth, respectively. Predictors with the suffix L1 denote their one-year lagged values, reflecting the influence of prior-year socioeconomic and governance conditions on current model outputs. SHAP values represent contributions to the model’s predicted IIM (per 100,000 population), and mean absolute SHAP values reflect average contribution magnitude across observations..

Figure 9 ranks variables by their mean absolute SHAP values and highlights distinct patterns in feature importance across the four sub-regions. Under the persistence-aware model, lagged intentional injury mortality dominates in most regions, confirming that historical mortality levels strongly influence current outcomes. This effect is particularly pronounced in South America (mean SHAP = 5.0) and Central America (mean SHAP = 6.0), where persistence is a defining characteristic of mortality dynamics. In North America, however, lagging unemployment emerges as the primary factor, indicating that prolonged labor-market instability has a greater structural impact than the still significant past IIM. The Caribbean also shows that lagged mortality has the highest feature importance in the persistence-aware model, while the lagged economic growth also contributes notably, implying that prior economic expansion helps buffer persistent mortality risk.

In contrast, the snapshot model prioritizes short-term influences: corruption leads in the Caribbean, inflation in Central America, and unemployment in North and South America. Collectively, these results reveal that persistence-aware modeling identifies enduring structural determinants that anchor regional mortality trajectories, whereas snapshot models emphasize the transient, contemporaneous pressures that trigger short-term fluctuations.

## Discussion

This study conceptualizes intentional injury mortality in the Americas as a complex outcome shaped by both immediate socioeconomic fluctuations and entrenched structural continuities. The findings, drawn from two complementary modeling frameworks, emphasize that short-term macroeconomic and governance disturbances coexist with long-term institutional and social inertia. The snapshot model captures contemporaneous (immediate) relationships, such as how current-year changes in unemployment, inflation, and corruption influence IIM. Meanwhile, the persistence-aware model includes lagged mortality and socioeconomic predictors to demonstrate the lasting “memory” of past conditions. Together, these models describe a dual dynamic: sudden economic and governance shocks quickly increase mortality risk, and their lasting effects extend this risk over future periods.

The results indicate that intentional injury mortality in the Americas reflects both immediate socioeconomic conditions and past influences. The snapshot model shows how current-year unemployment, inflation, and corruption align with current-year IIM, while the persistence-aware model incorporates lagged mortality and covariates, improving model fit across income categories. The exception is the upper-middle-income group, where the snapshot model exhibits relatively lower predictive performance. This reduced fit likely reflects the volatility and transitional nature of these economies, where labor market shocks, inflationary cycles, and governance shifts often occur simultaneously, blurring immediate causal relationships captured by contemporaneous predictors. In such settings, part of the macroeconomic effect is delayed, explaining why the persistence-aware model performs better when lagged adjustments are included. Overall, the figures show that short-run shocks matter and that their effects extend into subsequent periods, consistent with established links between economic strain, institutional quality, and violence or self-harm.

Income-stratified findings are internally coherent and align with the notion that development stage shapes the most salient near-term drivers. In high-income economies, corruption is the leading contemporaneous correlate of IIM. This is consistent with evidence that integrity losses reduce social trust and weaken the effectiveness of prevention and protection systems, allowing the same macroeconomic environment to convert into higher social risk^3,4^. In upper-middle-income economies, unemployment dominates; this accords with research showing that job loss and labor-market insecurity elevate psychosocial stress and community-level risk^8,9^. In lower-middle-income countries, GDP growth shows the clearest protective association, consistent with mechanisms where sustained expansion stabilizes expectations, supports employment, and increases fiscal capacity for social programs^17,70,71^.These directions are also visible in the SHAP summaries: corruption, unemployment, and inflation generally contribute positively to predicted IIM, while growth contributes negatively.

Building on the sub-regional findings reported in the Results section, distinct geographic patterns emerge across the Americas, particularly given that relatively few prior studies have examined these dynamics at this level.

Goodness-of-fit metrics should be interpreted in relation to modeling objectives and the underlying data structure. In cross-national macro-structural research characterized by heterogeneity and measurement variability, $\textrm{R}^{2}$ values ranging from 0.62 to 0.98, with most exceeding 0.80, reflect substantial predictive performance within a framework oriented toward prediction rather than parametric inference.^72^.

However, the model fit for North America is comparatively lower than that of other sub-regions, hinting that other factors may be at play, changing the dynamics, especially in North America, and affecting intentional injury mortality. In particular, factors such as firearm availability and access to healthcare and mental health services, which are known to shape homicide and suicide dynamics in the United States and Canada, are not explicitly included in the present macro-structural framework. The omission of these context-specific determinants may partially explain the comparatively lower predictive fit observed in North America. This leaves future researchers room for more scrutinized studies to unravel what’s missing in the current model to contrast with the rest of the American region.

South America shows strong positive contributions from inflation and unemployment, in both current and lagged forms, alongside clear gains from including persistence; this indicates that macroeconomic instability operates not only as an immediate pressure but also as a factor with carry-over effects into later periods. Central America displays a similar accumulation in the time dimension, with lagged unemployment and lagged inflation prominent in the persistence-aware model. The Caribbean presents a governance, centred profile, with corruption important in both specifications, consistent with regional findings linking institutional stress to elevated violence and self-harm risk^6,7^. Across these sub-regions, the persistence-aware model reduces residual error more than the snapshot model, and prediction-observation panels show narrower dispersion, especially at higher IIM values, which evidence that lagged conditions help explain levels and extremes.

The executed alignment in the study between the SHAP rankings and the change in fit across specifications strengthens the internal validity of the interpretation: temporal dependence is strongest where lagged features are most informative and weakest where current-year labor-market conditions explain the bulk of variation.

Moreover, the direction of effects is stable across most contexts and models in the study, allowing policymakers to assume that Unemployment, inflation, and corruption tend to increase predicted IIM, while GDP growth tends to decrease intentional injury mortality in the sub-regions of the Americas. These signs persist when moving from snapshot to persistence-aware formulations, while magnitudes and rank order adjust with the inclusion of lagged terms. For example, in North and South America, unemployment maintains a strong positive association in both models; in Central America, both unemployment and inflation retain positive directions with notable lagged contributions; in the Caribbean, corruption is consistently positive and comparatively large; in North America, unemployment is positive and leads both contemporaneously and with a lag. These regularities align with broader regional assessments that link macroeconomic volatility and governance quality to social harm^4,6,17^.

In the Caribbean, corruption stands out as the leading influence, pointing to the immediate role of governance quality. Central America is most affected by current inflation, reflecting how rising prices quickly translate into social and economic stress. In both North and South America, unemployment is the dominant factor, suggesting that real-time shifts in labor-market stability directly influence mortality risk. These differences indicate that while all regions face short-term socioeconomic pressures, the key immediate drivers vary according to each sub-region’s institutional and economic context^3,6,8,9^.

In light of the results of the current study, the persistence of intentional injury mortality remains strong across all income levels and most sub-regional investigations. There could most likely be some factors, such as underlying cultural, ethnic, religious, governmental structures, and societal long-term tragedies hidden beneath the sub-regional or country-level dynamics.^73,74^.

Furthermore, it is worth noting that North America differs from the rest of the region on three observable dimensions. First, in the persistence-aware model, lagged unemployment is more influential than lagged IIM, whereas in the Caribbean, South, and Central America, the lagged mortality term often carries the most significant persistence signal. Second, in the snapshot model for North America, current unemployment is the leading contemporaneous contributor. Third, the performance gain from adding persistence is smaller than in other sub-regions, indicating weaker temporal dependence of IIM on its own past. Read together, these points imply that labor-market conditions are the central measurable correlate in North America in both the current period and with a lag, while past mortality explains relatively less. This pattern aligns with evidence from advanced economies, where employment cycles strongly affect self-harm and violence outcomes. While the results are robust without requiring additional assumptions about unobserved drivers, future research may build on these findings by exploring deeper cultural and social dynamics to illuminate the underlying mechanisms further. However, the results are consistent with the idea that, in large high-income systems such as the United States and Canada, labor-market dynamics can dominate short-run risk and also leave a measurable imprint over time, while institutional persistence (as captured by lagged IIM) plays a comparatively lesser role.

The distinct North American pattern also becomes more intelligible when considered in light of the sub-region’s broader geographic, historical, and socioeconomic context. Comprising two advanced high-income economies included in the current study (Canada and the USA), North America is characterized by expansive mainland geography, diversified industrial structures, and highly cyclical labour markets that respond rapidly to global and technological fluctuations. Empirical analyses by the Macdonald-Laurier Institute indicate that official unemployment statistics in the United States and Canada often underestimate true labour-market slack, as discouraged and underemployed workers are not fully captured^75^. Consequently, increases in unemployment typically signal substantive socioeconomic strain rather than statistical noise. In addition, evidence also demonstrates that unemployment is strongly associated with substance-use disorders and mental-health deterioration, both established precursors of self-harm and interpersonal violence^76^. These mechanisms help explain why both current and lagged unemployment exerted dominant effects on intentional injury mortality within the North American models. Furthermore, the region’s liberal welfare regime, which continues to shape social policy design, offers comparatively limited social insurance and income support compared to Scandinavian countries model^77^. This structural characteristic allows labor-market shocks to produce both immediate and cumulative psychosocial consequences, sustaining elevated mortality risk even in the presence of strong institutions and economic recovery cycles.

There may also be deeper societal, cultural, and familial dynamics underlying the North American context, where unemployment intersects with gendered expectations, social isolation, and identity loss factors that can compound distress and, in some cases, manifest as both homicide and suicide. These patterns align with research linking economic marginalization and threatened masculinity to violent outcomes, underscoring that the relationship between unemployment and intentional injury mortality extends beyond economic deprivation to encompass broader psychosocial and cultural dimensions^78–81^.

Beyond interpretation, the explainable AI framework offers practical monitoring value. By tracking increases in lagged mortality contributions and shifts in dominant socioeconomic drivers across income levels and regional contexts, the model can signal when a system is entering a high-persistence risk phase. Such interpretable signals may support public health agencies and policymakers in initiating preventive interventions before elevated mortality patterns become structurally embedded.

Moreover, several limitations should be considered when interpreting the results. A key limitation concerns the operationalization of the dependent variable. In this study, intentional injury mortality was defined as the suicide–homicide composite in order to capture the overall macro-structural burden of intentional loss of life across countries and over time. However, homicide and suicide are not identical outcomes, and the same socioeconomic or governance-related factor may differ in direction, strength, or functional form across these two mortality types. As a result, the composite outcome may mask subtype-specific heterogeneity, and the present findings should be interpreted as relating to aggregate intentional injury mortality rather than to homicide or suicide individually. Furthermore, national averages can mask within-country differences in exposure, services, and institutions. The lag structure captures medium-run dependence but does not attribute persistence to specific micro-mechanisms (household, community, or institutional channels). The feature set is intentionally limited to broad macro-level regional factors due to data constraints for conducting large-scale regional studies. Other important factors, such as inequality, access to local services, exposure to violence, and cultural dynamics, may also be significant but are not examined in this analysis due to a lack of reliable, globally uniform indices.

Finally, the analysis focuses on the Americas, and transportability should be tested against local institutional and economic settings^82–84^. These limits are standard in regional mortality work and suggest that stronger national level data and additional covariates could sharpen mechanism identification. Overall, the evidence portrays intentional injury mortality as a temporally structured outcome whose immediate and lagged drivers are measurable and interpretable within an explainable-AI framework.

## Conclusion

The intentional injury mortality, which includes both homicide and suicide, has posed a persistent and rising threat to public health in the Americas over the past decade. The region continues to report some of the highest homicide rates globally and an upward trend in suicide. Unlike accidental injuries or many chronic diseases, these forms of mortality are not only deeply tragic but also largely preventable. Their intentional nature means they respond directly to policy, law, and structural interventions, such as firearm regulation, anti-corruption measures, mental health outreach, and community-based violence prevention. As such, IIM demands distinct analytical attention and a dedicated policy response.

This study advances the understanding of intentional injury mortality in the Americas by revealing that it is neither a purely contemporaneous nor an entirely historical phenomenon, but a hybrid process shaped by both immediate socioeconomic pressures and long-term structural inertia. The current study was conducted using income-level and sub-regional categorizations. Using explainable artificial intelligence, two complementary frameworks - snapshot and persistence-aware models jointly illuminate the mechanisms by which governance and macroeconomic factors sustain or mitigate violent mortality.

The model demonstrated excellent to acceptable performance across different income groups and sub-regions in this socioeconomic study. The persistence-aware model consistently demonstrated superior predictive performance across most income levels and sub-regions, confirming that once intentional injury mortality rises, its effects tend to endure through time. This persistence underscores that social, institutional, and psychological damage does not dissipate quickly even when surface-level economic conditions improve. Nevertheless, the snapshot model remains essential for capturing the immediate year-on-year sensitivity of intentional injury mortality to socioeconomic stress. Results showed that short-term fluctuations in unemployment, inflation, and corruption remain closely tied to contemporaneous changes in mortality. However, these short-term effects differ significantly across different income levels and sub-regions.

In high-income countries, corruption emerged as the leading contemporaneous factor, suggesting that governance integrity and institutional trust are decisive for public safety and mental well-being. In upper-middle-income economies, unemployment was most influential, consistent with prior research linking labor-market insecurity to social instability and self-harm. Lower-middle-income countries exhibited a stabilizing role for economic growth, suggesting that output and employment expansion provide a buffer against social strain. These findings collectively demonstrate that while mortality rate persistence is universal, the short-run triggers are context-dependent, underscoring the need for policies that align with each country’s economic and institutional capacity.

Sub-regional analysis reveal equally distinctive dynamics. South America and Central America exhibit strong mortality persistence effects, with lagged IIM, unemployment, and inflation all significantly predicting current mortality, suggesting that macroeconomic instability becomes embedded in social outcomes over time. The Caribbean displays a governance-driven pattern dominated by corruption, aligning with regional evidence that weak institutions perpetuate both violence and self-harm.

In contrast, North America diverges from these trends: lagged unemployment rather than lagged intentional injury mortality emerged as the main persistence factor, while current unemployment remained the leading contemporaneous driver. This dual influence of labor market stress indicates that in large high-income systems such as the United States and Canada, intentional injury mortality dynamics are more responsive to employment cycles than to institutional continuity. The relatively lower performance in model fit for North America further suggests that additional factors, such as mental health access, inequality, or cultural variation may play a greater role there than the present variables capture.

Empirical evidence supports the potential impact of such interventions. In Europe, active labor market policies such as training programs and direct job creation schemes, alongside passive income support during long-term unemployment, have been associated with reductions in suicide mortality^85^. In the United States, minimum wage increases have been linked to measurable declines in non-drug suicides among low-educated adults, illustrating how wage stabilization policies can mitigate deaths of despair^86^. On the institutional side, Boston’s Operation Ceasefire under the focused deterrence framework demonstrated how coordinated enforcement and credible rule implementation reduced youth homicide^87^. Similarly, the so-called “Bogotá miracle” of the 1990s has been attributed to judicial strengthening, anti-corruption reforms, and enhanced enforcement certainty that contributed to substantial declines in homicide rates^88^. Together, these cases illustrate how labor-market stabilization and institutional integrity reforms can translate into tangible reductions in intentional injury mortality.

In contexts where mortality risk escalates rapidly, linking Public Health Emergency Declarations to automatic social security disbursements may provide immediate economic stabilization, particularly during inflationary spikes or unemployment shocks. Such coordinated policy triggers could buffer vulnerable populations and reduce the likelihood that short-term socioeconomic distress evolves into persistent intentional injury mortality patterns. In conclusion, this research reframes intentional injury mortality as a multidimensional and temporally persistent outcome that requires dual-horizon policy making. Short-term responses should focus on mitigating immediate economic and governance shocks by stabilizing employment, implementing anti-corruption initiatives, and providing targeted financial protection during inflationary periods. Long-term strategies must address structural and psychological persistence by strengthening institutional integrity, promoting equitable economic growth, and expanding sustained mental-health infrastructure. Future research should integrate subnational data and policy-event timelines to assess how institutional reforms or economic interventions modify persistence patterns. By combining AI-driven interpretability with economic reasoning, this study offers an empirically grounded framework for understanding how societies accumulate and sustain risk and how deliberate institutional renewal can ultimately reduce it.

## Supplementary Information

Supplementary Information 1.

Supplementary Information 2.

## Data Availability

All data used in this study are publicly accessible through the WHO Mortality Database and the World Bank Open Data portal. These datasets can be obtained directly from the following links: https://www.who.int/data/data-collection-tools/who-mortality-database and https://data.worldbank.org/.
