# Supplementary material for: Explainable AI for public health surveillance: investigating the persistent crisis of intentional injury mortality (suicide and homicide) in the Americas
Source: Sci Rep. 2026 May 24;16:23762. doi: 10.1038/s41598-026-51327-y (PMC13429623; doi:10.1038/s41598-026-51327-y)
Supplement: Supplementary file 1 — Supplementary Information 1. [file 41598_2026_51327_MOESM1_ESM.pdf]

Multicollinearity Diagnostics Report
(Including Lagged Dependent Variable)

Dependent variable: IIM

Lagged dependent variable: IIM_L1 (IIM at t−1)

Structural predictors: INFL, UNEMP, CORR, EG

A) Data Summary

Total rows in file (after year parsing): 500

Rows used for diagnostics (complete IIM_L1 + predictors): 475

Detected panel identifier (country/location): Country (unique: 25)

Detected time variable: Year (range: 2000–2019)

1) VIF and Tolerance (Including IIM_L1)

Rule of thumb: VIF < 5 (no serious multicollinearity), 5–10 (moderate), > 10 (severe).
Tolerance < 0.1 often indicates serious multicollinearity.

Variable	VIF	Tolerance
IIM_L1	1.1691	0.8554
INFL	1.1233	0.8903
UNEMP	1.1070	0.9034
CORR	1.2383	0.8076
EG	1.0797	0.9262

2) Correlation Matrix (IIM_L1 + Predictors)

Pearson correlations computed on complete cases for IIM_L1 and predictors.

Variable	IIM_L1	INFL	UNEMP	CORR	EG
IIM_L1	1.0000	-0.0200	0.1210	-0.3198	-0.0364
INFL	-0.0200	1.0000	0.1815	-0.1754	-0.1566
UNEMP	0.1210	0.1815	1.0000	0.1061	-0.1733
CORR	-0.3198	-0.1754	0.1061	1.0000	-0.1363
EG	-0.0364	-0.1566	-0.1733	-0.1363	1.0000

3) Correlation Between Lagged IIM and Structural Predictors

This section directly addresses collinearity concerns between IIM(t−1) and structural predictors.

Variable	Corr(IIM_L1, X)
INFL	-0.0200
UNEMP	0.1210
CORR	-0.3198
EG	-0.0364

4) High Pairwise Correlations (|r| ≥ 0.80)

No pairs exceeded |r| ≥ 0.80.
