# Supplementary material for: Explainable AI for public health surveillance: investigating the persistent crisis of intentional injury mortality (suicide and homicide) in the Americas
Source: Sci Rep. 2026 May 24;16:23762. doi: 10.1038/s41598-026-51327-y (PMC13429623; doi:10.1038/s41598-026-51327-y)
Supplement: Supplementary file 2 — Supplementary Information 2. [file 41598_2026_51327_MOESM2_ESM.pdf]

Supplementary Table S1: Comprehensive comparison of predictive performance across all evaluated state-of-the-art machine learning models, including RMSE, MSE, R^2, MAE, and MAPE metrics used to identify the optimal model for the main analysis.

ML Model	Validation					Test				
	RMSE	MSE	R2	MAE	MAPE %	MAE	MSE	RMSE	R2	MAPE %
Linear	10.02	100.34	0.58	7.87	35.98	8.92	163.8	12.8	0.48	36.53
Interactions Linear	8.03	64.48	0.73	5.75	26.83	8.37	114.79	10.71	0.63	36.08
Robust Linear	10.19	103.81	0.56	7.9	37.25	9.14	166.62	12.91	0.47	38.12
Stepwise Linear	8.42	70.88	0.7	6.1	28.6	7.92	104.38	10.22	0.67	34.33
Fine Tree	8.13	66.15	0.72	5.21	22.29	4.04	48.2	6.94	0.85	16.27
Medium Tree	7.69	59.14	0.75	5.35	22.52	5.65	76.3	8.74	0.76	22.6
Coarse Tree	10.28	105.73	0.56	7.74	31.85	9.07	153.03	12.37	0.51	34.85
Linear SVM	10.49	110.05	0.54	7.73	36.31	9.57	193.21	13.9	0.39	39.81
Quadratic SVM	7.02	49.22	0.79	4.41	20.38	5.38	39.73	6.3	0.87	24.99
Cubic SVM	11.05	122	0.49	5.34	22.9	4.12	31.91	5.65	0.9	18.76
Fine Gaussian SVM	11.53	132.89	0.44	8.68	34.51	6.9	101.12	10.06	0.68	30.19
Medium Gaussian SVM	5.81	33.75	0.86	3.95	17.56	5.3	40.21	6.34	0.87	23.56
Coarse Gaussian SVM	10.74	115.42	0.51	7.64	28.39	9.28	195.95	14	0.38	31.91
Efficient Linear Least Squares	10.02	100.33	0.58	7.87	35.98	8.92	163.8	12.8	0.48	36.53
Efficient Linear SVM	12.99	168.86	0.29	9.13	34.06	10.26	205.84	14.35	0.34	38.07
Boosted Trees	6.56	43.07	0.82	4.75	19.51	5.31	64.61	8.04	0.79	20.92
Bagged Trees	7.33	53.75	0.77	5.27	21.74	5.25	50.91	7.14	0.84	20.91
Squared Exponential GPR	5.91	34.92	0.85	3.99	17.89	4.44	35.32	5.94	0.89	19.85
Matern 5/2 GPR	5.87	34.47	0.85	3.83	17.41	4.45	35	5.92	0.89	20.07
Exponential GPR	6.1	37.21	0.84	3.81	17.58	4.69	38.21	6.18	0.88	20.78
Rational Quadratic GPR	5.94	35.29	0.85	3.88	17.67	4.51	35.67	5.97	0.89	20.32
Narrow Neural Network	8.99	80.85	0.66	5.16	22.45	4	26.65	5.16	0.92	18.71
Medium Neural Network	11.2	125.39	0.47	6.22	27.57	4.15	32.44	5.7	0.9	18.14
Wide Neural Network	10.92	119.21	0.5	7.17	31.01	6.88	73.14	8.55	0.77	36.13

Bilayered Neural Network	10.77	115.98	0.51	6.07	25.74	4.98	46.04	6.79	0.85	22.75
Trilayered Neural Network	10.57	111.62	0.53	6.69	29.36	6.33	67.28	8.2	0.79	26.9
SVM Kernel	11.97	143.25	0.4	7.94	25.89	9.11	177.87	13.34	0.43	31.19
Least Squares Regression Kernel	6.1	37.22	0.84	4.57	20.9	6.3	65.6	8.1	0.79	27.56
TabPFN	1.60	2.56	0.98	1.3	4.5	1.46	3.1	1.76	0.98	5.2

Supplementary Table S2: Hyperparameter configurations used for each machine learning model during performance benchmarking, supporting full reproducibility of the comparative experiments.

ML Model	Hyperparameters
Linear	Terms: Linear; Robust option: Off
Interactions Linear	Terms: Interactions; Robust option: Off
Robust Linear	Terms: Linear; Robust option: On
Stepwise Linear	Initial terms: Linear; Upper bound on terms: Interactions; Maximum number of steps: 1000
Fine Tree	Minimum leaf size: 4; Surrogate decision splits: Off
Medium Tree	Minimum leaf size: 12; Surrogate decision splits: Off
Coarse Tree	Minimum leaf size: 36; Surrogate decision splits: Off
Linear SVM	Kernel function: Linear; Kernel scale: Automatic; Box constraint: Automatic; Epsilon: Auto; Standardize data: Yes
Quadratic SVM	Kernel function: Quadratic; Kernel scale: Automatic; Box constraint: Automatic; Epsilon: Auto; Standardize data: Yes
Cubic SVM	Kernel function: Cubic; Kernel scale: Automatic; Box constraint: Automatic; Epsilon: Auto; Standardize data: Yes
Fine Gaussian SVM	Kernel function: Gaussian; Kernel scale: 0.5; Box constraint: Automatic; Epsilon: Auto; Standardize data: Yes
Medium Gaussian SVM	Kernel function: Gaussian; Kernel scale: 2; Box constraint: Automatic; Epsilon: Auto; Standardize data: Yes
Coarse Gaussian SVM	Kernel function: Gaussian; Kernel scale: 8; Box constraint: Automatic; Epsilon: Auto; Standardize data: Yes
Efficient Linear Least Squares	Learner: Least squares; Solver: Auto; Regularization: Auto; Regularization strength (Lambda): Auto; Relative coefficient tolerance (Beta tolerance): 0.0001;

Efficient Linear SVM	Learner: SVM; Solver: Auto; Regularization: Auto; Regularization strength (Lambda): Auto; Relative coefficient tolerance (Beta tolerance): 0.0001; Epsilon: Auto
Boosted Trees	Minimum leaf size: 8; Number of learners: 30; Learning rate: 0.1; Number of predictors to sample: Select All
Bagged Trees	Minimum leaf size: 8; Number of learners: 30; Number of predictors to sample: Select All
Squared Exponential GPR	Basis function: Constant; Kernel function: Squared Exponential; Use isotropic kernel: Yes; Kernel scale: Automatic; Signal standard deviation: Automatic; Sigma: Automatic; Standardize data: Yes; Optimize numeric parameters: Yes
Matern 5/2 GPR	Basis function: Constant; Kernel function: Matern 5/2; Use isotropic kernel: Yes; Kernel scale: Automatic; Signal standard deviation: Automatic; Sigma: Automatic; Standardize data: Yes; Optimize numeric parameters: Yes
Exponential GPR	Basis function: Constant; Kernel function: Exponential; Use isotropic kernel: Yes; Kernel scale: Automatic; Signal standard deviation: Automatic; Sigma: Automatic; Standardize data: Yes; Optimize numeric parameters: Yes
Rational Quadratic GPR	Basis function: Constant; Kernel function: Rational Quadratic; Use isotropic kernel: Yes; Kernel scale: Automatic; Signal standard deviation: Automatic; Sigma: Automatic; Standardize data: Yes; Optimize numeric parameters: Yes
Narrow Neural Network	Number of fully connected layers: 1; First layer size: 10; Activation: ReLU; Iteration limit: 1000; Regularization strength (Lambda): 0; Standardize data: Yes
Medium Neural Network	Number of fully connected layers: 1; First layer size: 25; Activation: ReLU; Iteration limit: 1000; Regularization strength (Lambda): 0; Standardize data: Yes
Wide Neural Network	Number of fully connected layers: 1; First layer size: 100; Activation: ReLU; Iteration limit: 1000; Regularization strength (Lambda): 0; Standardize data: Yes
Bilayered Neural Network	Number of fully connected layers: 2; First layer size: 10; Second layer size: 10; Activation: ReLU; Iteration limit: 1000; Regularization strength (Lambda): 0; Standardize data: Yes
Trilayered Neural Network	Number of fully connected layers: 3; First layer size: 10; Second layer size: 10; Third layer size: 10; Activation: ReLU; Iteration limit: 1000; Regularization strength (Lambda): 0; Standardize data: Yes
SVM Kernel	Learner: SVM; Number of expansion dimensions: Auto; Regularization strength (Lambda): Auto; Kernel scale: Auto; Epsilon: Auto; Standardize data: Yes; Iteration limit: 1000
Least Squares Regression Kernel	Learner: Least Squares Kernel; Number of expansion dimensions: Auto; Regularization strength (Lambda): Auto; Kernel scale: Auto; Standardize data: Yes; Iteration limit: 1000

Supplementary Table S3: Comparison of Mean SHAP Values between TabPFN and Medium Gaussian SVM

Income_Level	Feature	TabPFN_SHAP	Med_Gauss_SVM_SHAP	Difference
Level 1	CORR	0.65	0.58	0.07
	UNEMP	0.51	0.48	0.03
	INFL	0.38	0.32	0.06
	EG	0.38	0.35	0.03
Level 2	CORR	1.00	0.92	0.08
	UNEMP	2.50	2.15	0.35
	INFL	0.45	0.40	0.05
	EG	0.40	0.42	0.02
Level 3	CORR	0.01	0.05	0.04
	UNEMP	0.05	0.10	0.05
	INFL	0.01	0.04	0.03
	EG	1.41	1.28	0.13
